# Recent Progress in the Pathology and Genetics of Pilocytic and Pilomyxoid Astrocytomas

**DOI:** 10.4274/balkanmedj.2018.1001

**Published:** 2019-01-01

**Authors:** Cristine Ding, Tarik Tihan

**Affiliations:** 1 Division of Neuropathology, Department of Pathology, UCSF School of Medicine, California, USA; 2 Department of Pathology, Tan Tock Seng Hospital, Novena, Singapore

**Keywords:** Astrocytoma, glioma, mitogen-activated protein kinase pathway, oncogene-induced senescence, pilocytic, pilomyxoid

## Abstract

Pilocytic and pilomyxoid astrocytomas are some of the most common gliomas in children and young adults. These gliomas are indolent neoplasms with long overall survival probability. The genetic characteristics of these neoplasms are well known, and our deepened understanding of their associated molecular alterations has led to the development of novel treatment strategies and approaches. Currently, we can account for some of the unusual behavior, such as oncogene-induced senescence, associated spontaneous regression, anaplastic transformation, and cerebrospinal dissemination, of these gliomas. Nevertheless, enigmatic issues continue to surround these chronic tumors. Here, we review the classical and uncommon clinical pathological and genetic features of these indolent gliomas.

Pilocytic astrocytomas (PAs) are the most common primary gliomas in children and adolescents (0-19 years of age). A recent CBTRUS report stated that PAs account for approximately 15.6% of brain tumors (1). In the United States, the annual incidence of PAs is approximately 0.35-0.37 per 100.000 persons. This incidence peaks among children aged 0-9 years and decreases with advancing age (1).

Bailey and Cushing (2) historically referred to PAs as “spongioblastoma unipolare”. Subsequently, Penfield and his colleagues from the Montreal Neurological Institute introduced the term “piloid (hair-like) astrocytes” to describe astrocytes with long parallel hair-like fibers that are found in areas of gliosis (3). The term “pilocytic astrocytoma” was later used by Russell and Bland (4) and was officially used in the 1979 WHO classification (5). The current WHO classification defines PA as a WHO grade I neoplasm (6).

PA often presents indolent behavior and has excellent prognosis with overall survival rates of more than 20 years. Given these characteristics, PA is considered as a “chronic disease”, a neoplasm that requires a tempered approach and long-term follow-up (7). However, except for the recently described PA variant, pilomyxoid astrocytoma (PMA), a small percentage of PAs exhibits aggressive behavior that may not be predictable upon histological examination (8).

Considerable information about PA, including its defining genetic aberrations, potential for spontaneous regression, unpredictable radiation response, and anaplastic transformation, has been gleaned over the past decade. In this review, we present our current understanding of the histopathologic, radiologic, and molecular aspects of PA and its variant, PMA. We also discuss critical issues, such as prognostically relevant parameters, that may provide insight into the biological behaviors of these gliomas.

## Clinical Considerations

PA can occur anywhere within the neuraxis. In children, they usually arise in the posterior fossaand less commonly in the spinal or supratentorial regions. Among adults, the incidences of PA in infra- and supratentorial locations negligibly differ (9). PAs can arise in association with neurofibromatosis type 1 (*NF1*), wherein they most commonly manifest as “optic glioma,” a nonspecific term that implies the involvement of the optic nerve proper (10). Progression-free and overall survival probabilities are influenced by several factors, including the location of the tumor and the extent of resection, and may be modified by access to healthcare (11). Patients who undergo the gross total resection of their tumors have excellent prognosis (12), whereas those with deeply situated lesions and who receive subtotal resections may experience recurrence (13). Yet, even subtotally resected tumors may not progress over time. Overall, PAs are slow-growing indolent neoplasms with 10-year survival rates that exceed 95% (9).

An interesting and widely reported feature of PA is its propensity to undergo spontaneous regression (14,15). This phenomenon can be observed after subtotal resection and in the absence of any adjuvant treatment. The biological mechanisms underlying tumor regression remain poorly elucidated. Moreover, the spontaneous regression of PA cannot be accurately predicted. Oncogene-induced senescenc edriven by mitogen-activated protein kinase (MAPK) may play a major role in the tumor regression and long-term survival of patients with PA.

The clinical presentation of PA directly reflects its location. Patients with posterior fossa tumors generally present with slowly progressive headaches, nausea, vomiting, or ataxia, as well as other signs and symptoms of cerebellar dysfunction. Cerebral tumors often present with focal signs peculiar to the location of the tumor. Tumors involving the hypothalamus and adjacent visual pathways can cause visual loss or field defects, as well as symptoms and signs related to hypothalamic-pituitary dysfunction, e.g. obesity, diabetes insipidus, ordiencephalic syndrome (16). Tumors involving the thalamus and basal ganglia can cause contralateral weakness. Seizures are exceptional even with cerebral tumors. Tumors of the optic nerve can cause visual loss and may induce proptosis. Spinal cord tumors can cause signs of cord compression. Infrequently, patients may present with acute neurological deficits due to intratumoral hemorrhage (17,18,19), which may present a diagnostic challenge because its rapid clinical course and complex appearance on magnetic resonance imaging (MRI) are suggestive of a high-grade neoplasm or a vascular malformation. Rarely, PA, particularly PMA, may present with evidence of dissemination or cerebrospinal fluid spread (8,20,21,22,23).

## Radiologic Features

**Figure 1:**
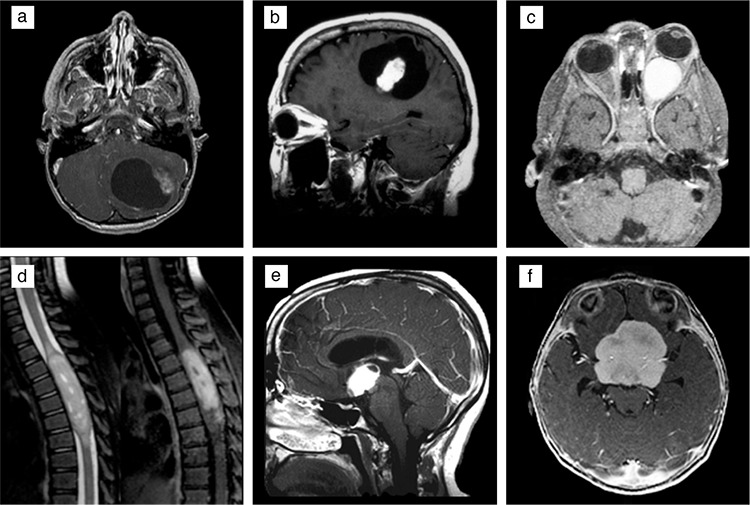
Radiologic features of pilocytic and pilomyxoid astrocytoma on magnetic resonance imaging. Pilocytic astrocytomas displaying typical circumscribed, cystic appearances with enhancing mural nodules in the (a) cerebellum and (b) supratentorial region. Expansion of the left optic nerve by a solid, enhancing pilocytic astrocytomas (c). T2-weighted and contrast-enhanced images from an intramedullary pilocytic astrocytomas involving the spinal cord (d). Mainly solid, enhancing pilocytic astrocytomas with minimal cystic component involving the hypothalamic region (e). Lobulated, solid enhancing pilomyxoid astrocytoma involving the hypothalamic region (f). All figures are contrast-enhanced T1 weighted images, with the exception of the one-dimensional T2-weighted image showing a spinal cord tumor.

PA is typically a well-circumscribed, cystic mass with a contrast-enhancing intramural nodule and little/no peritumoral edema or mass effect (Figure 1). Tumor calcification may be occasionally recognized on computed tomography or MRI. On MRI, the nodule is usually iso-or hypointense to gray matter on T1-weighted images and hyperintense on T2-weighted images. Some cases may exhibit the distortion of the fourth ventricle and associated hydrocephalus. The radiological designation of “exophytic” tumor in the brainstem is often associated with PA. However, this designation is often used too loosely to be of specific significance. Tumors of the optic nerve are typically solid without a cystic component, cause the expansion and distortion of the nerve, and may result in the “dotted i” appearance of the intraorbital optic nerve by causing the buckling of the nerve just proximal to the globe (Figure 1c). Hypothalamic tumors are mostly solid (Figure 1e). PMA typically occurs in the hypothalamus, particularly in young children. In the supratentorial region, PA scan arise adjacent to the third ventricle and show a typical cystic and solid appearance (Figure 1b) (24,25).

Similar to PA, PMAs are most commonly located in the hypothalamic/chiasmatic region, although they can occur in other locations in the neuraxis (26). However, in contrast to PA, they are predominantly solid and rarely have cystic components (27,28). Some hypothalamic/chiasmatic PMAs appear as solid, bulky masses (Figure 1f) and may extend into the temporal lobes (24). Almost all PMAs are well circumscribed and show little or no peritumoral edema (29). On MRI, they are typically hypointense on T1-weighted images and hyperintense on T2-weighted images (6,28). They show homogeneous or heterogeneous contrast enhancement (28,29). Intratumoral hemorrhage has been reported in up to 25% of tumors in one series; this rate is higher than that reported for PAs (26). Radiologic evidence of cerebrospinal fluid dissemination tends to be more common in PMA than in PA (23).

## Histological Features

Classically, PAs are noninfiltrating tumors that display low to moderate cellularity and a characteristic biphasic pattern with varying proportions of compact, bipolar cells with hair-like fibrillary processes. Most PAs harbor Rosenthal fibers within their compact or fibrillary areas. Although the presence of Rosenthal fibers in compact or fibrillary areas is a highly helpful feature in recognizing PA, it is not an absolute requirement for diagnosis. Another caveat with Rosenthal fibers is that their presence in the reactive glial tissues that surround other indolent tumors may mislead the observer to consider PA. Tumors, such as pineal parenchymal tumors, craniopharyngiomas, and hemangioblastomas, as well as ependymomas of the spinal cord, often incite reactive gliosis and Rosenthal fiber formation in the adjacent neuropil, sometimes leading to the misrecognition of the tumor as PA.

**Figure 2:**
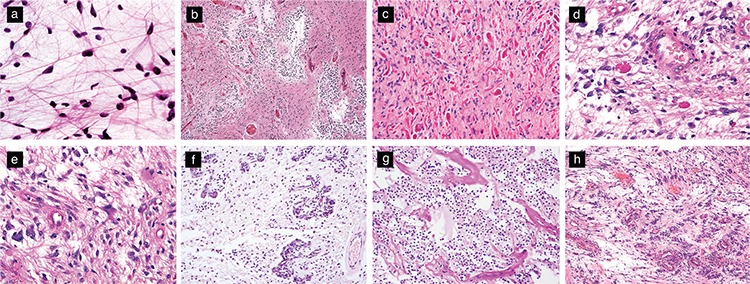
Histologic features of pilocytic astrocytomas. Bipolar cells with hair-like cytoplasmic processes that are best visualized with cytologic smears (H&E, original magnification 400×) (a). Classic biphasic appearance featuring alternating compact and loose areas associated with microcystic spaces (H&E, original magnification 100×) (b). Compact areas containing numerous Rosenthal fibers (H&E, original magnification 200×) (c). Eosinophilic granular bodies (H&E, original magnification 200×) (d). Enlarged multinucleated cells with a “pennies on a plate” arrangement (H&E, original magnification 200×) (e). Glomeruloid vascular proliferation (H&E, original magnification 200×) (f). Oligodendroglioma-like region (H&E, original magnification 200×) (g). Polar spongioblastoma-like pattern (H&E, original magnification 200×) (h).

A typical histological appearance of PA is that of loose areas with multipolar cells associated with microcysts. This pattern combined with focal myxoid (or mucinous) changes sometimes invokes the diagnosis of “microcystic astrocytoma,” an arcane term typically used for diffuse astrocytomas in the cerebrum. Eosinophilic granular bodies may also be present but are often fewer than Rosenthal fibers (Figure 2a-2d). Notably, the biphasic pattern is seen in only a proportion of cases and is thus not essential for diagnosis (30). Some tumors, especially those of the cerebellum, may show areas that resemble oligodendroglioma (Figure 2g), whereas others may show a polar spongioblastoma-like pattern that demonstrates a step-ladder formation characterized by nuclear palisades (Figure 2h) (30,31). Cytologically, the cells display uniform, bland nuclei, although some cells with hyperchromatic and pleomorphic nuclei unaccompanied by increased mitoses may be present. Degenerative atypia that feature pleomorphic nuclei and nuclear-cytoplasmic inclusions and enlarged cells with multiple nuclei arranged peripherally (Bernd W. Scheithauer’s “pennies on a plate” arrangement) may also be present (Figure 2e) (6). PAs are highly vascular tumors and can present a linear arrangement of glomeruloid vascular proliferation (Figure 2f) that outlines the cystic space, as well as thick-walled hyalinized vessels and glomeruloid type proliferative capillaries in a linear arrangement along the cyst wall (30). Hemorrhage and regressive angiomatous vascular changes may be sufficiently prominent to simulate vascular malformation. The presence of nonpalisading, infarct-like necrosis is rare but has not been associated with aggressive behavior (32). Rare mitotic figures may be present. Similar to numerous low-grade neoplasms, such as gangliogliomas or pleomorphic xanthoastrocytomas, PA can spread into the subarachnoid space and extend through Virchow-Robin spaces. In such cases, the tumor cells attain a spindled morphology and may appear different from those in the intraaxial component; nevertheless, this feature has not been associated with aggressiveness (6,30).

PAs usually show a variable degree of infiltration into the adjacent brain parenchyma despite radiologic circumscription. Some rare examples of PAs within the cerebellum present a diffuse microscopic appearance (the “diffuse PAs” of Bernd W. Scheithauer) while retaining typical compact or microcystic patterns at least focally and show similar prognosis as tumors with classical histological features (33,34). These tumor cells can infiltrate the adjacent parenchyma over distances of a few millimeters to centimeters but generally do not aggressively overrun the neuropil (6). Limited or small biopsies from these regions may cause diagnostic difficulty and misdiagnoses, e.g. as oligodendroglioma or diffuse astrocytoma.

**Figure 3:**
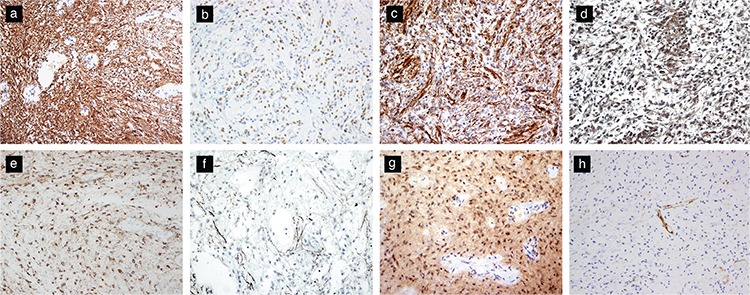
Immunohistochemical features of pilocytic astrocytomas. PAs typically show diffuse positive staining for GFAP (original magnification, 200×) (a), Olig-2 (original magnification, 200×) (b), and Vimentin (original magnification, 200×) (c). Synaptophysin expression (original magnification, 200×) (d) and MAP-2 expression (original magnification, 200×) may be present (e). Neurofilament stain highlighting a limited infiltrative pattern at the periphery of the tumor (original magnification, 200×) (f). BRAFV600e mutant protein expression is seen in rare tumors with the mutation (original magnification, 200×) (g). Loss of p16 staining may portend poor prognosis (original magnification, 200×) (h).

Immunohistochemical features of PA are helpful in their recognition. One of the most practically useful stains is the neurofilament protein that typically highlights the non-infiltrative or solid nature of the tumor. One exception to this is the so-called diffuse pattern and the limited biopsies from the periphery of the tumor, where the solid nature of the tumor may not be readily recognized. The tumors are often strongly and diffusely positive with GFAP, Vimentin, Olig-2 (Figure 3a-c) and S100 protein, as well as the transcription factor SOX-10 (6,11,35,36). Absence of GFAP, Olig-2 and S100 staining is unusual and should lead one to question the diagnosis of PA. Many PAs demonstrate diffuse, albeit weak staining with Synaptophysin but even focal strong positive staining can be encountered (Figure 3d). Another neural antibody, MAP-2, can also be positive (Figure 3e) and may be focally strong in PA, while the neuronal antibody Neu-N is typically negative (11,35). Ki67 proliferation index is low in most cases. P53 is staining is weak to absent, but even weak positive staining tumors do not harbor *TP53* mutations. Immunostaining for *BRAF V600E*-mutant protein may be positive in a rare tumor with this mutation (Figure 3g), but more often the antibodies against this mutant protein are not helpful in the interpretation (6). Tumors with the typical molecular composition also stain diffusely and strongly with the p16 antibodies and tumors that stain entirely negatively with this antibody may suggest a more aggressive behavior (Figure 3h). Many PAs are also positive with the WT-1 antibody. While often not of any practical utility, staining with SOX2, NSE and CD56 can be positive in PA. CD34 is typically negative in tumor cells and highlight vasculature, a feature that differentiates PA from glioneuronal tumors such as ganglioglioma. Where available, pERK staining often correlates with the presence of MAPK pathway activation often associated with the typical *BRAF* duplication (11).

The designation of anaplasia in PA and the characterization of anaplastic PA as a distinct variant or a different entity entirely remain highly controversial. Nevertheless, anaplastic features in some PAs have been well documented (“PA with anaplasia”) despite their rarity (6). Anaplastic features may be observed at first presentation or in recurrent tumors with or without adjuvant treatment. The vast majority of PAs show morphologic stability even upon recurrence with only exceptional tumors exhibiting progression to anaplastic histology (9,37). Anaplastic features have been seen in only 1.7% of 2200 PA cases (38). The histologic features of anaplasia include hypercellularity, moderate to severe cytologic atypia, and brisk mitotic activity (>4 mitoses per 10 high-power fields) with or without necrosis (6,38). The anaplastic component may display fibrillary, small cell, or epithelioid morphology, as well as increased Ki-67 labeling index, but most often the labeling index shows areas with low values typical of PA. Strong p53 staining has been reported in rare anaplastic examples, but whether this staining corresponds to a *TP53* mutation remains unclear (38).

A considerable proportion of tumors with anaplasia have occurred in the setting of prior radiation therapy (38,39) even though anaplastic transformation is not always associated with a history of prior irradiation (37,38,40). This transformation can also occur in patients with *NF1* (38,41). PAs with anaplasia are associated with poor median overall survival and progression-free survival compared with those associated with typical PA but better survival than that associated with diffuse high-grade gliomas. Patients with survival beyond 5 or 10 years have been reported (9,42). Given that anaplastic PAs are not aggressive, most authors avoid using the term “glioblastoma ex PA” (6,38). Anaplastic examples of PAs have not received a WHO grade in the most recent WHO classification scheme (6).

## Pilomyxoid Variant

PMA is the only recognized variant of PA. The term “pilomyxoid” was first introduced in 1999 (8) and was subsequently codified as a WHO Grade II tumor in the 2007 WHO (43). These tumors tend to occur in young patients and appear to show a less favorable prognosis than PA because they exhibit local recurrence and cerebrospinal spread (8,22). The true incidence of PMA is unknown given that they are often overlooked and sometimes over diagnosed among PAs. An original study in 1999 identified 18 patients from a large cohort of 1013 patients with PAs, giving a rate of approximately 1.7%. A single-institution study found that PMA accounts for approximately 10% of cases previously diagnosed as PA within a 13-year period (44). Given variations in diagnostic criteria, a strong possibility exists that some studies overestimate the prevalence of PMA among pilocytic neoplasms.

**Figure 4:**
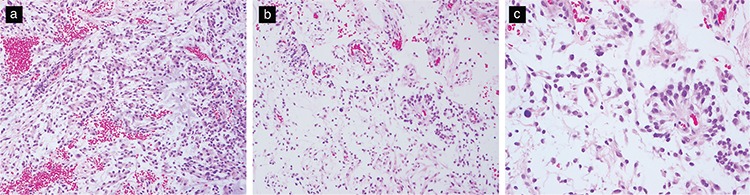
Histological features of PMA. Monomorphous bipolar tumor cells in an expansive myxoid matrix (H&E, original magnification 200×) (a,b). Tumor cells arranged in a radiating angiocentric pattern around vessels (H&E, original magnification 400×) (c).

Histologically, PMAs display a marked myxoid matrix and monomorphous bipolar cells that often radiate from vessels in an angiocentric pattern (Figure 4). Typically, the tumors are compact and noninfiltrative but may display infiltration and trap normal brain elements similar to those displayed by PA. PMAs lack Rosenthal fibers and a biphasic pattern and exceptionally demonstrate rare eosinophilic granular bodies. Mitotic figures may be present and are often more easily recognized compared with those in typical PA. Akin to PA, vascular proliferation often occurs in the form of linear glomeruloid tufts associated with the cyst wall. Rarely, some PMAs demonstrate focal, nonpalisading necrosis (6,22).

Features supportive of PMA as a variant of PA include the younger ages of patients with PMA compared with those of patients of PA (22), examples of differentiation or “maturation” from pilomyxoid to pilocytic morphology in recurrent tumors (45,46,47), and tumors showing “intermediate” pilomyxoid features. The latter has been well recognized and may actually be as common as the pure PMA variant, and the biological behavior of these “hybrid” tumors are not entirely clear (47). Given that focal pilomyxoid changes in an otherwise typical PA have not been associated with poor prognosis, these neoplasms may not warrant the designation of PMA (6). Nevertheless, commenting on the presence and extent of pilomyxoid features may be prudent because their exact behavior remains unclear (47).

The grading designation of PMA was removed from the 2016 WHO classification because these tumors have similar molecular/genetic characteristics. This unfortunate assessment was based on the uncertain interpretation of the biological behavior of PMA. Regardless of the decision regarding the WHO classification scheme, substantial evidence—and probably more robust evidence than many other entities in the current classification with a designated grade—shows that PMA follows a more aggressive course than PA even when controlled for age and location (48). Based on the available data in the literature, substantial evidence favors the characterization of PMA as a more aggressive and higher grade variant of PA, as demonstrated in well-designed and well-characterized cohort studies (22).

**Figure 5:**
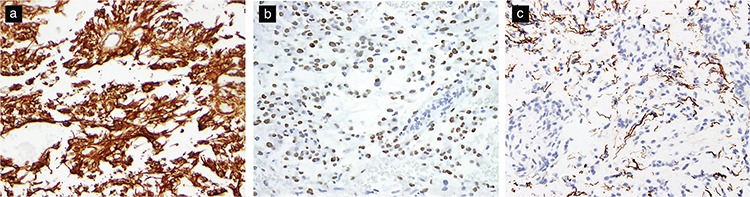
Immunohistochemical features of pilomyxoid astrocytoma. Positive staining for GFAP (original magnification, 200×) (a) and Olig-2 (original magnification, 400×) (b). Neurofilament stains may highlight an infiltrative pattern at the tumor periphery (original magnification, 200×) (c).

The immunohistochemical staining features of PA are virtually identical to those of PMA (Figure 5). PMAs are also positive for GFAP, S100 protein, vimentin, Olig-2, and SOX10, and some cases express synaptophysin (6,36,47). Staining for neurofilament and chromogranin-A are typically negative, or the former can show focal or limited axonal staining (6). A range of Ki-67 labeling index has been reported, but it is generally low (8,46). The Ki-67 labeling indexes of PMA and PA considerably overlap (6).

## Molecular and Genetic Characteristics

The association of PA with NF1 has been recognized for many years, and PA constitutes one of the most common types of gliomas in patients with NF1. PMAs have also been reported in some patients with NF1 (26). The NF1 germline mutation is inherited in an autosomal dominant manner, but approximately half of all patients have a sporadic or *de novo* *NF1* mutation (6). The NF1 gene is located on chromosome 17q11.2 and encodes for the gene product neurofibromin, a GTPase-activating protein that functions as a negative regulator of the RAS protein in the mitogen MAPK pathway, which mediates cell growth, survival, and differentiation. The loss of the NF1 protein results in the activation of cell proliferation pathways and decreases cell differentiation through the activation of the MAPK pathway or alternatively through the activation of mammalian target of rapamycin (mTOR) pathway (6,49).

**Figure 6:**
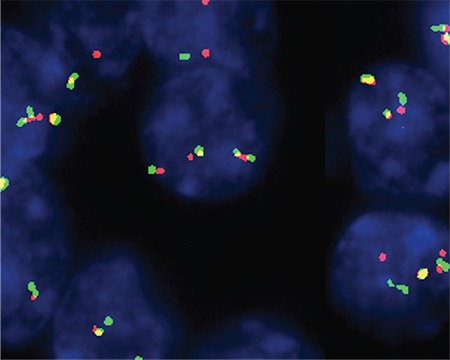
*KIAA1549-BRAF* fusion can be detected through fluorescence in situ hybridization. In this example, a yellow fusion signal is seen in most of the tumor nuclei.

Information about the genetic and molecular characteristics of non-*NF1*-associated PA or PMA remained limited until approximately a decade ago when several independent studies found that the majority (>70%) of PA cases demonstrated the presence of an oncogenic tandem duplication at 7q34. This duplication results in a *KIAA1549-BRAF* fusion gene (Figure 6) with constitutive *BRAF* kinase activity and MAPK pathway activation. *KIAA1549-BRAF* fusions are found at all anatomical locations but are more frequently found in cerebellar tumors than in other sites. Multiple differentexonic fusion combinations between *KIAA1549* and *BRAF* have been reported but all result in the loss of the *BRAF* autoregulatory N-terminal domain and the retention of the *BRAF* kinase domain; these effects subsequently result in the constitutive activation of the oncogenic MAPK pathway (50,51,52). The frequency of *KIAA1549-BRAF* fusion is low in adult tumors (79% of tumors diagnosed in the first decade of life compared with 30% of patients aged 31-40 years and 7% of patients older than 40 years) (53). A small number of cases with other *BRAF* fusion gene partners have also been found, including *FAM131B*, *RNF130*, *CLCN6*, *MKRN1*, *GNA11*, *QKI*, *FXR1*, and *MACF1* (6,54).

The abnormalities of genes encoding for proteins with influences on the MAPK pathway have been detected in almost all PAs and most PMAs (6).The second most common alteration is a *BRAF* hotspot mutation that results in a valine to glutamate substitution at position 600 (*BRAF V600E* mutation). This mutation has been found in approximately 10% of PA cases and frequently in PAs inextra-cerebellar locations, such as diencephalic tumors. This mutation is commonly found in other gliomas such as pleomorphic xanthoastrocytomas and gangliogliomas (55). Other alterations of the components of the MAPK pathway include *FGFR1* mutation and fusions/duplication, *NTRK2* fusions, *SRGAP3-RAF1* fusion, *KRAS* mutation, and *PTPN11* mutation (51,56,57). These MAPK pathway alterations appear to be largely mutually exclusive, with only rare reports of concurrent *BRAF V600E* mutation and *KIAA1549-BRAF* fusion (35,58).

**Figure 7:**
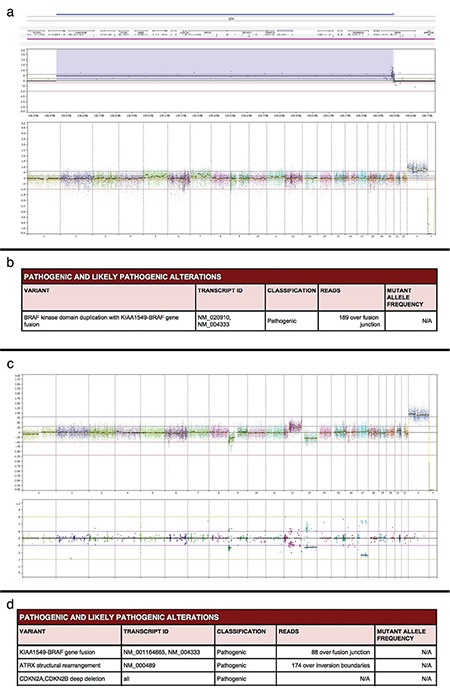
Capture-based next-generation sequencing findings for pilocytic astrocytoma: Genome-wide copy number profile revealing a focal copy number gain on chromosome 7q34 that involves the *BRAF* gene without additional chromosomal gains, losses, or focal amplifications or deletions in a typical pilocytic astrocytoma (a). Final analysis confirming the presence of *KIAA1549-BRAF* gene fusion in the tumor (b). Example of an anaplastic pilocytic astrocytoma showing multiple chromosomal copy number changes, including the gain of 12q and the losses of 1p, 9p, and 13 (c). Anaplastic pilocytic astrocytoma demonstrating *KIAA1549-BRAF* gene fusion as well as an *ATRX* structural rearrangement and *CDKN2A* and *CDKN2B* deletions (d).

The *KIAA1549-BRAF* fusion gene was initially thought to be highly specific for PAs, especially in tumors that virtually lack copy number variations or other mutations (Figure 7). However, rare examples of low-grade glioneuronal tumors, diffuse astrocytomas, oligodendrogliomas, and glioblastoma have been subsequently reported (50,59,60,61,62). Interestingly, while in some of these cases, the diagnoses are quite contentious and may be challenged, almost all tumors with the *KIAA1549-BRAF* fusion have histological components that appear to be typical for PA. For example, gangliogliomas in the posterior fossa (63) demonstrated two genetic subtypes, one of which had a typical PA component and the *BRAF* fusion. Recent studies on the gangliogliomas of the CNS also identified rare cases of gangliogliomas with a pilocytic component having the identical *KIAA1549-BRAF* fusion gene as those of PAs (Melike Pekmezci, personal communication). Nevertheless, the presence of *KIAA1549-BRAF* fusion and absence of other molecular genetic alterations remain consistent with the diagnosis of PA in the appropriate morphologic and clinical setting.

Conflicting reports on the influence of *KIAA1549-BRAF* fusion on the behavior of PA or PMA exist. Several studies found that clinical outcome is independent of *BRAF* status (59,64,65), whereas others have found that fusion is an independent prognostic marker for significantly improved 5-year progression-free survival (58). One study has reported that disseminated PAs possess genetic features similar to those of classic PA, with a similar incidence of *KIAA1549-BRAF* fusions and *BRAF* mutations (66).

Approximately 60% of PMA cases harbor *KIAA1549-BRAF* fusions (58), whereas only the rare *BRAF V600E* mutation has been reported in these tumors (67). Gene expression microarray studies have shown differences in gene expression between PMA and PA, whereby PMAs overexpress the developmental genes *H19* and *DACT2*, extracellular matrix collagens (COL2A1; COL1A1), and IGF2BP3. However, how these genes influence the biological behavior of PMA remains unclear given that tumors with similar microarray profiles demonstrate diverse clinical outcomes (46).

Oncogene-induced senescence (OIS) refers to a process in tumors whereby the arrest of tumor growth is triggered in response to oncogene overexpression. *In vitro* studies have demonstrated that MAPK pathway activation using constitutively active *BRAF* induces the initial proliferation of PA cells, followed by OIS and the cessation of cellular proliferation (68,69). The induction of OIS by MAPK pathway activation due to solitary genetic alterations may be the underlying reason for the slow growth, indolent behavior, and/or spontaneous regression of PA. OIS may be mediated by the p16 protein (*CDKN2A*) given that in immortalized astrocytes, the loss of this protein abrogated OIS (69). Consistent with this hypothesis, patients with p16-negative tumors have significantly poorer outcomes than those without (68,70). Further more, the loss of p16 has been reported in some anaplastic PA (42,71), and a recent study involving 73 anaplastic astrocytomas with pilocytic features found that these gliomas are characterized by frequent MAPK pathway alterations, *CDKN2A/B* deletion, *ATRX* loss, and unfavorable prognosis (72). Notably, some treatments, such as radiotherapy, may abrogate OIS through the introduction of additional mutations or gene silencing within crucial checkpoints, such as *CDKN2A* and p53, and may then be associated with adverse outcomes (35).

Other genetic alterations that appear to adversely affect prognosis in PA include whole chromosome 7 gain and heterozygosity loss on 17p13, both of which are associated with an increased risk of recurrence (64,65). In addition, a recent meta-analysis of the *H3K27M* mutation in circumscribed (non-diffuse) gliomas included seven PAs with *H3K27M* mutation, these being associated with a poorer outcome than *H3K27M* wild-type circumscribed gliomas but better prognosis than diffuse gliomas with or without the mutation (73). The PI3K/AKT-mTOR pathway may also play a role in these tumors, with increased PI3K/AKT being reported in PA, particularly those with anaplasia (41,74). However, none of these studies provide conclusive evidence and convincing data that would constitute grounds to utilize these markers in the clinical diagnosis of PA or PMA.

Surgical resection is the preferred treatment for tumors at sites where gross total or subtotal resection can be achieved. However, adjuvant therapy may be required for patients with residual unresectable tumors or those with disease progression and/or dissemination. The discovery of MAPK and mTOR pathway alterations have led to the use of novel targeted therapeutic agents. Multiple ongoing clinical trials use *BRAF* (ClinicalTrials.gov: NCT01677741 and NCT01748149), MEK (ClinicalTrials.gov: NCT01089101), and mTOR inhibitors (ClinicalTrials.gov: NCT01734512) as targeted agents for gliomas and other tumors that harbor mutations. These studies also use combinations of these drugs to circumvent cellular escape mechanisms and drug resistance, e.g. *BRAF* inhibitor da*BRAF*enib with MEK inhibitor trametinib (ClinicalTrials.gov: NCT02124772).

Caution should be exercised in the selection of these therapies given that our understanding of the complex molecular interactions that affect the MAPK pathway and its related pathways remains incomplete. This challenge was illustrated by an earlier experience with sorafenib, a multikinase inhibitor that targets *BRAF*, VEGFR, PDGFR, and c-kit. A multicenter phase II study conducted to determine the response rate to sorafenib in patients with recurrent or progressive pediatric low-grade gliomas (ClinicalTrials.gov: NCT01338857) was terminated early because of the unexpectedly rapid and high tumor progression rate. Subsequently, *in vitro *studies indicated that this effect is likely related to paradoxical ERK activation (75). Therapies should be as specific as possible to address the underlying molecular alterations within each tumor to minimize bystander effect. Comprehensive histological analysis and the identification of specific molecular/genetic abnormalities of the tumor and the interaction with the host should be critical in understanding the response and outcome of individual patients.

PA tumors have heterogeneous morphologies and behaviors. As a group, they show predominantly excellent prognosis. However, accurately predicting the behavior of individual PA tumors is difficult given that tumors with similar histology and molecular genetic alterations behave differently. Our understanding of the biology of these tumors has greatly improved with recent molecular genetic discoveries, which have also led to new options for targeted therapy. However, the complex interactions that occur among altered pathways in these tumors remain to be fully elucidated. We hope that future research will provide a complete understanding of the drivers that initiate oncogenesis and that factors that lead to progression of these tumors to enable the development of effective therapies. Notably, PA and PMA are mostly indolent tumors and may exhibit a protracted clinical course and regression. Therefore, we recommend caution in treatment and avoid overtreatment, particularly in younger patients.
